# Cononsolvency Transition of Polymer Brushes: A Combined Experimental and Theoretical Study

**DOI:** 10.3390/ma11060991

**Published:** 2018-06-11

**Authors:** Huaisong Yong, Sebastian Rauch, Klaus-Jochen Eichhorn, Petra Uhlmann, Andreas Fery, Jens-Uwe Sommer

**Affiliations:** 1 Leibniz-Institut für Polymerforschung Dresden e.V., 01069 Dresden, Germany; yong@ipfdd.de (H.Y.); Rauch@ipfdd.de (S.R.); kjeich@ipfdd.de (K.-J.E.); uhlmannp@ipfdd.de (P.U.); 2 Institute of Physical Chemistry of Polymeric Materials, Technische Universität Dresden, 01062 Dresden, Germany; 3 Institute for Theoretical Physics, Technische Universität Dresden, 01062 Dresden, Germany

**Keywords:** cononsolvency, preferential adsorption, polymer brushes, PNiPAAm, ellipsometry

## Abstract

In this study, the cononsolvency transition of poly(*N*-isopropylacrylamide) (PNiPAAm) brushes in aqueous ethanol mixtures was studied by using Vis-spectroscopic ellipsometry (SE) discussed in conjunction with the adsorption-attraction model. We proved that the cononsolvency transition of PNiPAAm brushes showed features of a volume phase transition, such as a sharp collapse, reaching a maximum decrease in thickness for a very narrow ethanol volume composition range of 15% to 17%. These observations are in agreement with the recently published preferential adsorption model of the cononsolvency effect.

## 1. Introduction

Cononsolvency occurs if a mixture of two good solvents causes the collapse and segregation of polymer solutions into a polymer-rich phase in a certain range of compositions of these two solvents [1,2]. A prominent example [3] is poly(*N*-isopropylacrylamide) (PNiPAAm) in a water and methanol mixture. Although both solvents are good solvents for PNiPAAm, in a certain range of compositions of methanol and water PNiPAAm segregates from the mixed solution. Besides segregation in solutions, the cononsolvency-induced volume phase transition of PNiPAAm gels has received much attention since the first paper published by T. Amiya et al. [4]. It is notable that a discontinuous, or jump-like, volume phase transition as it is observed for gels in such solvent mixtures would not be expected for a transition from a good to poor solvent within the common Flory-Huggins scheme because of the absence of translational degrees of freedom of the chains here [5,6]. This indicates a new kind of transition due to the solvent–cosolvent–polymer interaction. Given these observations, it is interesting to explore the possibility of a jump-like collapse transition for polymers on surfaces, in particular for polymer brushes.

Controlling the swelling of the polymer brushes and achieving a switching effect is quite important for the study of phase transitions [7,8,9,10,11] and applications of polymer brushes [12,13,14]; however, study of cononsolvency effects as a new switching effect in polymer brushes is still in its infancy stage. On the side of the experimental studies, previous studies mainly focused on how to use modern measurement techniques to monitor the cononsolvency transition of brushes. Liu and Zhang [15] used a quartz crystal microbalance with dissipation monitoring (QCM-D) to study the behavior of PNiPAAm brushes in water-methanol mixtures. They found a sharp cononsolvency transition, which they attributed to the formation of water-methanol complexes or clusters via hydrogen bonding. Later, S. Edmondson et al. [16] directly observed the volume change of polymer brushes by using ellipsometry to investigate the cononsolvency transition of poly(2-(methacryloyloxy)ethyl phosphorylcholine) brushes in alcohol-water mixtures. X. Sui et al. [17] observed morphological changes of PNiPAAm brushes by using surface probe microscopy in methanol-water mixtures. By using an extended surface forces apparatus, R. Espinosa-Marzal et al. [18] studied the impact of solvation on equilibrium conformation of dextran brushes in aqueous dimethyl sulfoxide mixtures. By combining the methods mentioned above, cononsolvency effects of polymer brushes have been related to applications for nanomaterials [19], tunable friction [20,21] and adhesive [22] properties of surfaces, as well as response of polymer brushes in liquid fluid media [23,24].

A possible molecular explanation for the new transition comes from atomistic simulations of single chains in the presence of a cosolution [25], and has been rationalized using the concept of preferential and non-specific adsorption of a cosolvent onto the polymer chain [26,27]. Based on the concept of preferential adsorption, one of the authors proposed a general model for a cononsolvency transition in polymer brushes [28], which has been extended recently to polymer solutions [29]. Chen et al. [30] also proposed a polymer lattice density functional theory (PLDFT) to investigate the cononsolvency phenomena related to polymer adsorption in a slit pore.

What is missing up to now is a physical understanding of the cononsolvency effect on real polymer brushes, which explains the equilibrium behavior and nature of the phase transition. The aim of this work is an investigation on the cononsolvency transition behavior in hydrophilic polymer brush systems in comparison with the theoretical predictions. We especially focus on a systematical VIS-spectroscopic ellipsometry (SE) measurement to study the properties of PNiPAAm brushes grafted on flat silicon surfaces in a mixture of ethanol and water.

## 2. Materials and Methods

Poly (glycidyl methacrylate) (PGMA, *M_n_* = 10–20,000 g/mol) was purchased from Sigma-Aldrich (Darmstadt, Germany). Absolute ethanol (EtOH, 99.8%) was acquired from Merck (Darmstadt, Germany). Tetrahydrofuran (THF, 99.98%) and chloroform (CHCl_3_, ≥99%) were purchased from Acros Organics (Darmstadt, Germany). Purified water (H_2_O) was used from a Milli-Q Direct-8 system from Merck Millipore (Darmstadt, Germany). All chemicals were used as received, if not otherwise specifically noted. Highly polished single-crystal silicon wafers of {100} orientation (Si-Mat Silicon Materials, Kaufering, Germany) were used as a substrate. End-functionalized PNiPAAm polymers (*M_n_* = 50,200 g/mol, *M_w_/M_n_* = 1.28, monomer number of every chain is about 443) with a terminal (*tert*-butyl protected) carboxy group were synthesized and characterized as described previously [31].

### 2.1. Preperation of Polymer Brushes

**Scheme 1:**
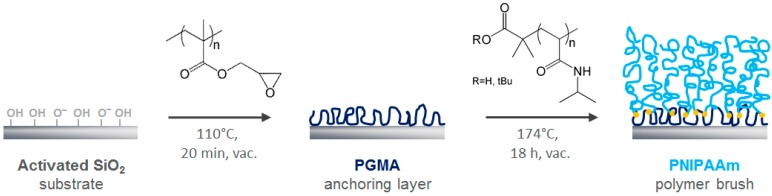
Preparation of PNiPAAm brushes on silicon substrates using the “grafting-to” approach.

Silicon substrates (13 × 20 mm^2^) were treated with EtOH in an ultrasonic bath for 10 min, dried with a stream of nitrogen, and treated with oxygen plasma (440-G Plasma System, Technics Plasma GmbH, Wettenberg, Germany) for 1 min at 100 W. Next a thin layer of PGMA (2.5 nm) was deposited by spin coating (Spin 150, SPS Coating, Ingolstadt, Germany) a PGMA solution in CHCl_3_ (0.3 mg/mL) with subsequent annealing at 110 °C in a vacuum oven for 20 min to react the silanol groups of the substrate with a fraction of the epoxy groups of PGMA, thus forming an anchoring layer equipped with the remaining epoxy groups for the following “grafting-to” process [32]. Afterwards, a filtered solution of end-functionalized PNiPAAm in THF (9.0 mg/mL) was spin coated onto the PGMA layer and subsequently annealed at 174 °C in a vacuum oven for 18 h(Scheme 1). To remove non-covalently bonded polymers, the resulting films were immersed first in H_2_O, then extracted in H_2_O overnight, rinsed with EtOH, and dried in a stream of nitrogen.

Here we point out the primary advantage of the “grafting-to” method is that it is a technically simple processing step consuming low quantities of an accurately characterized pre-formed polymer. This means molecular weight, polydispersity, chain–end functionality, and especially the architecture can be designed very precisely, and thus comprehensive studies like the present one can be carried out.

### 2.2. VIS-Spectroscopic Ellipsometry Measurement

A spectroscopic ellipsometer (alpha-SE, Woollam Co., Inc., Lincoln, NE, USA) equipped with a rotating compensator was used to measure the relative phase shift (Δ) and the relative amplitude ratio (tan Ψ) of the polymer brush films in the dry state, as well as in-situ in purified H_2_O and H_2_O/EtOH mixtures within a batch cuvette (TSL Spectrosil, Hellma, Muellheim, Germany) at constant temperature of 25 °C [33]. All solvent mixtures were prepared in the same day or one day before measurements and sealed in glass vials at room temperature. We always use the same brush by rinsing the solvent and introduce another solvent with help of a syringe-pump apparatus. The cuvette was flushed at least three times with the current solvent mixture right before measurement to avoid changes in the composition of the ambient. All measurements were performed between 400 and 800 nm at an angle of incidence Φ_0_ of 70°, which is close to the Brewster angle of silicon. To evaluate the index of refraction and thickness of the brush films in dry (*h*) state and in situ (*H*), a multilayer-box-model consisting of silicon, silicon dioxide, anchoring layer PGMA, and a polymer brush was assumed [31]. The refractive indices of the environment (water and H_2_O/EtOH mixtures) were measured using a digital multiple wavelength refractometer (DSR-lambda, Schmidt + Haensch, Berlin, Germany). All data was acquired and analyzed using the Complete EASE^®^ software package (version 4.46). For the brush sample used in this work, we did three-time repeatable ellipsometry measurements. Result differences of these measurements were small, and as such, data reproducibility of our ellipsometry measurements were quite good.

From the determined film thickness (*h*), polymer brush parameters like grafting density (*σ*) and distance between anchoring points (*S*) can be calculated by using Equation (1):

*σ* = *S*^–2^ = *N_A_ρh*/*M_n_*, (1)

where *M_n_* is the number-average molecular weight of the polymer, *N_A_* is the Avogadro’s number, *ρ* is the polymer’s melt density [34]. Here, *ρ* is 1.1 g/cm^3^ for PNiPAAm homo-polymer. For the interpretation of our measurements we have to assume a lateral homogeneous polymer profile. This is the case for the brush regime of strongly overlapping chains. To determine whether the grafted polymers are in the brush regime, the distance between grafting sites, *S,* should be small enough. For the case of a good solvent, *S* should be much smaller than twice the radius of gyration of the chains (*R_G_*):

*S*/2 < *R_G_* = *αN*^0.588^, (2)

where *α* is a prefactor proportional to the monomer size and *N* is the degree of polymerization; for the PNiPAAm polymer, *α* is estimated to be 0.3 nm [35,36]. However, in the collapsed state, this condition is not sufficient. Here, so-called octopus-micelles can be formed if the grafting density is below the stretching threshold of σ** [37],

(3) $$\sigma^{**}=N_{A}\rho \alpha /(M_{0}\sqrt{N}),$$

where *M_0_* is the molecular weight of repeat units of the polymer. If *σ* > σ**, the chains will form stretched brushes. This condition gives the upper estimate for the grafting density, which is necessary for a homogeneous brush. One might also use the overlap radius of gyration of the chains (*R_P_*) between collapsed single chains as a rough lower estimate for a homogeneous brush under the condition that chains collapse mostly, i.e., *R_P_* = α*N*^1/3^ and *S*/2 < *R_P_*, where the radius of gyration under poor solvent conditions is taken. We note that this estimate slightly underestimates the necessary grafting density. Basic physical parameters of PNiPAAm brushes used in this study are listed in the Table 1.

## 3. Results and Discussion

### 3.1. Ellipsometry Analysis of the Cononsolvency Transition in PNiPAAm Brushes

**Figure 1:**
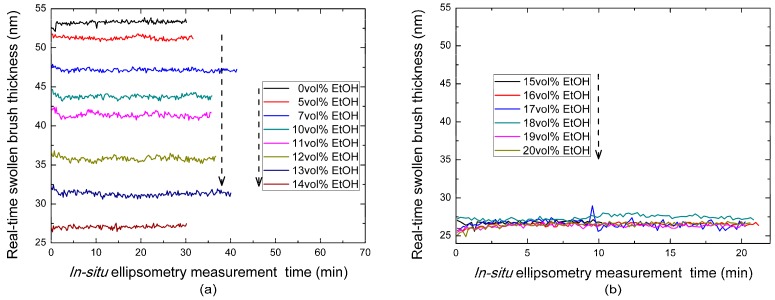

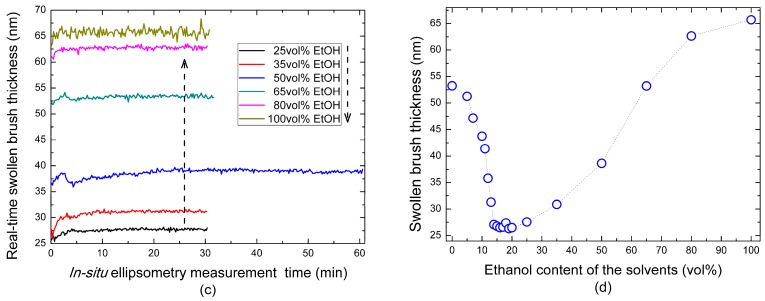
Swollen brush thickness of a PNiPAAm brush (*σ* = 0.20 chains/nm^2^) in aqueous ethanol mixtures as a function of (**a**–**c**) measurement time and (**d**) ethanol fraction obtained via Vis-spectroscopic ellipsometry. Note that the dashed line in Figure 1d is only used to guide eyesight. The error bars in Figure 1d are neglected because they are quite small (around 0.2 nm).

Although previous experimental studies [15,16,17,18,19,20,21,22,23,24] claimed that the cononsolvency transition of polymer brushes show features of a phase transition, so far, direct experimental evidence is missing. In order to check whether the cononsolvency transition of polymer brushes is an equilibrium transition, we measured swollen brush thickness as a function of time. Typical real-time measurements of V is-spectroscopic ellipsometry for swollen brush thickness of PNiPAAm are shown in Figure 1a–c. Except for the condition where the ethanol volume fraction is around 50%, it is clearly seen that after about 5 min of continuous measurement, the brush system became stable. For the condition of the ethanol volume fraction around 50%, in current measurement, the time needed to wait for the brush system to become stable was more than 20 min. Thus, the data shown in Figure 1a–c supported the equilibrium nature of the cononsolvency transition that we report in this work.

Here, we point out that these differences in waiting time to reach equilibrium state may depend on the polymer characteristics itself and subsequent response. As the PNiPAAm polymer is quite thermally sensitive, even the enthalpy change via changing solvent composition can lead to changes in the polymer’s configuration [38]. We also observed that with an increasing ethanol fraction, especially for the regime of high ethanol concentration, scatter in the real-time brush thickness increased. This might be explained due to lower optical contrast between the ambient ethanol and the thin PNiPAAm film. Note that all data about swollen brush thickness in the following are averaged values of real-time ellipsometry data in the stable regime, except when specifically indicated.

**Scheme 2:**
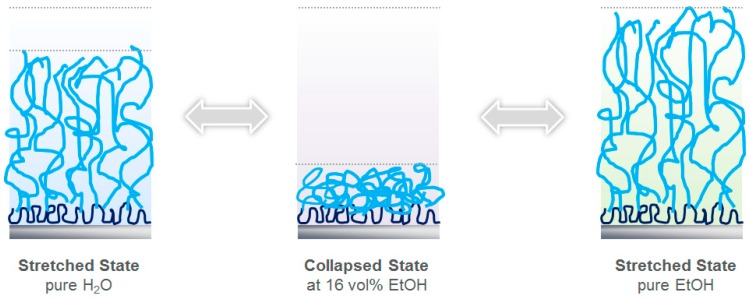
Main cononsolvency transition regimes of a PNiPAAm brush in H_2_O/EtOH mixtures.

Figure 1d summarises results for the PNiPAAm brush thickness (please see raw data in Appendix A.1) as a function of ethanol volume fraction (V_E_). We were able to qualitatively distinguish five solvent composition regimes: (i) the brush thickness of PNiPAAm was hardly affected by the change of V_E_ when it was lower than 0.05; (ii) for the range of 0.05 < V_E_ < 0.15, there was a sharp collapse transition of brushes, reaching a maximum decrease of thickness in a very narrow composition range of 0.15 ≤ V_E_ ≤ 0.17 (Scheme 2); (iii) for the range of 0.17 < V_E_ < 0.3, there was no obvious change of brush thickness in this regime; (iv) for the range of 0.3 < V_E_ < 0.8, there was a re-entrant transition of brushes from a collapsed state to a swollen state; and (v) for the range of V_E_ > 0.8, the brushes were fully re-swelling in ethanol-rich solvents. The brush thickness in pure ethanol (V_E_ = 1) was always thicker than in pure water (V_E_ = 0). This was a direct consequence of the stronger attraction between the alcohol and the monomer as compared to water and the monomer. This can be related with a larger excluded volume coefficient, and thus to stronger swelling. We also note that this phenomenon could be partially attributed to the molecular volume of ethanol being larger than the water’s (Scheme 2, please see more details in the Appendix A.2).

In previous studies, Y. Yu et al. [21] also measured swollen thicknesses of PNiPAAm brushes with respect to a composition change of ethanol. Different from our results, they concluded that there was a non-sharp re-entrant transition of brushes by using ellipsometry measurements, reaching a maximum decreasing of thickness in a very broad composition range of 0.2 < V_E_ < 0.3. The ellipsometry analysis of Reference [21] showed that the difference in swollen brush thickness in pure water and ethanol is negligible, which is in contrast with the corresponding AFM (Atomic force microscopy) measurement. Our own experience indicated that these discrepancies may be due to insufficient equilibration of the brush system in their ellipsometry measurements. Nevertheless, their AFM measurement results of the composition range [21] for the collapse transition with increasing ethanol concentration are consistent with our ellipsometry measurement results. We note that the grafting density in Reference [21] was chosen to be higher than in experiments reported here. According to the adsorption-attraction model, as discussed below, this should lead to an increase of the ratio between the height of the collapsed state and the height of the swollen state in pure water (deswelling ratio), which can indeed be concluded from the AFM measurement results of Reference [21].

**Figure 2:**
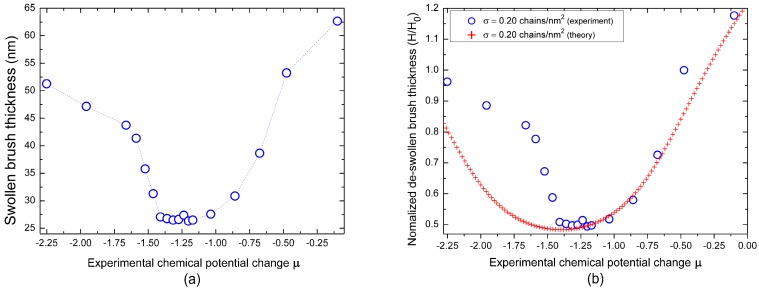
(**a**) Cononsolvency transition of a PNiPAAm brush (*σ* = 0.20 chains/nm^2^) in water/ethanol mixtures with reference to the swollen brush thickness as a function of experimental chemical potential change. Note that the dashed line is only used to guide eyesight. (**b**) Theoretical fitting of normalized de-swollen brush thickness plotted with respect to experimental chemical potential change in cononsolvency transition of a PNiPAAm brush.

Merely based on the results presented in Figure 1d, one should not conclude that the “swelling-to-collapse” transition with increasing ethanol concentration was sharper than the “collapse-to-swelling” transition with increasing ethanol concentration. Indeed, after transforming ethanol volume fraction (V_E_) into experimental chemical potential change, *μ* (please see more details in the Appendix A.3), it is clearly shown in Figure 2a that this “swelling-to-collapse-to-swelling” re-entrant transition curve became close to symmetric. According to the recently published adsorption-attraction model [28], the chemical potential change of solvent mixtures, not the volume fraction change of solvent mixtures, determines the equilibrium state of the brush. In particular, a nearly symmetric behavior of the brush thickness for collapse and reentry transitions is expected from the adsorption-attraction model [28].

### 3.2. Application of the Adsorption-Attraction Model for Cononsolvency Transition in PNiPAAm Brushes

To theoretically analyze the experimental results, we use the recently proposed adsorption−attraction model [28], which is based on the concept of preferential adsorption of a cosolvent onto the polymer as proposed by atomistic simulations [27]. The adsorption−attraction model predicts the free energy expression of the brush–solvent–cosolvent system using the equations listed below [28]:

*f*(*φ*, *c*) = *f_ads_* + *f_attr_* + *f_brush_*, (4)

*f_ads_* = *φ*ln*φ* + (1 − *φ*)ln(1 − *φ*) − *μφ* − *εφ*, (5)

*f_attr_* = −2*εγφ*(1 − *φ*)*c*, (6)

*f_brush_ = tσ*^2^/(2*c*^2^) *+* (1/*c* − 1 − *νφ*)ln(1 − *c* − *νφc*). (7)

Here, *f*(*φ*, *c*) is the free energy per monomer in the brush layer, where *c* is the volume fraction of monomers in the brush layer. The first term, *f_ads_*, corresponds to the adsorption of an adsorbed cosolvent on the monomers of the chains. The number of cosolvent molecules per monomer is denoted by *φ*. The preferential adsorption is expressed by the energy gain *ε* per adsorbed cosolvent. The first two terms in *f_ads_* in Equation (4) correspond to the lattice gas state of a cosolvent on the polymer chains as a substrate. The chemical potential change of the solvent and cosolvent is denoted as *μ*.

The term *f_attr_* denotes the mean-field attraction between monomers caused by forming a bridge due to the cosolvent. The strength of this additional attraction is given by *γε*, which takes into account bridging and size effects of the cosolvent by considering *γ* ≠ 1. The last group of terms, *f_brush_*, corresponds to the conformational free energy of the brush as a function of the volume fraction of monomers and of the grafting density, *σ*. The first term in *f_brush_* is the free energy for stretching, *t* is a numerical prefactor, which accounts for the specific conformations of chains in a polymer brush diverging from Alexander-de Gennes approach; the second term is the Flory-Huggins free energy per monomer of the brush. Here, we took into account the effective increase of volume by adsorption of a cosolvent, where *ν* denotes the added volume fraction by full saturation of the polymer by a cosolvent. We note that no explicit interactions between the three components, other than the adsorption of a cosolvent on the chains, is considered. If taken pairwise, all components are fully miscible, and the collapse of the brush can only be the result of a cosolvent-induced attraction between the monomers.

The minimum for the free energy in Equation (4) can be obtained analytically for the “symmetric case” *ν* = 0. Here, one can show that the effect of the cosolvent can be mapped to an effective concentration-dependent χ-parameter. As a consequence, the brush collapse can be of first order for a certain range of grafting densities and solvent selectivities. It is worth noting that the degradation of quality of a simple solvent cannot give rise to a step-like collapse transition because of the absence of translational entropy in this case. For the adsorption-attraction model, one can show that the effective attraction between monomers is also affected by the higher-order virial coefficients. Here, a coexistence between an effective 3-monomer attraction and an effective 2-monomer repulsion led to the first-order transition scenario. We note that experimental data indicated such as strong collapse behavior in the range of 15–20% volume fraction of ethanol. For more details about the nature of the collapse/demixing transition due to preferential cosolvent adsorption we refer the reader to References [28,29].

The relation between the volume fraction of monomers in the brush layer and the height of the brush is *H* = *σN*/*c*. The equilibrium height of the brush is given by the minimization of Equation (4) with respect to both free variables *c* and *φ*. We display the numerical result together with the measurement data as a function of the chemical potential. Parameter values used in our theoretical fitting are *t* = 0.04, *ν* = 0.07, *γ* = 1, and *ε* = 1.25. Note that in Figure 2b, *H_0_* is the swollen thickness of PNiPAAm brushes in pure water.

Based upon our theoretical fitting in Figure 2b, it was shown that the adsorption–attraction model can give a good description for a collapsed state of cononsolvency transition of polymer brushes. By way of qualitative analysis, it should be that the competition between attraction energy penalty of *f_attr_* and energy gains of *f_ads_* and *f_brush_* controls the “sharpness” of the cononsolvency transition; however, a closed analysis of the model with respect to the experimental results can only be done if essential parameters, such as grafting density or chain length, can be varied. Moreover, the exact value of the chemical potential difference in the mixed solvent is most important for a precise fitting since a shift of *μ* is related with a shift in the unknown solvent–solvent selectivity parameter *ε*, and thus changes the sharpness of the transition. We note that taking into account additional effects of the interaction between the components can lead to refinement of the prediction, in particular, the location and strength of the collapse transition. It has already been pointed out in previous studies that interaction between the solvent and cosolvent could play a role in the cononsolvency transitions of polymer brushes [15,18]. We also note that the effect of polydispersity is not taken into account in Equation (4). Without doubt, polydispersity plays an important role in affecting properties of polymer brushes [39,40,41].

## 4. Conclusions

In the present study, we investigated the cononsolvency effect of PNiPAAm brushes with moderate grafting density in aqueous ethanol mixtures. We have used Vis-spectroscopic ellipsometry measurements to prove the hypothesis that the cononsolvency transition of PNiPAAm brushes consists of a volume phase-like transition. For low ethanol fractions in the system, there was a sharp collapse of the brushes, reaching a minimum in film thickness in a very narrow ethanol volume fraction range from 15% to 17%. This was followed by a reentry transition at a volume fraction in the range of 30% to 80%. If the chemical potential is taken as the control variable, both transitions were qualitatively similar, while the reentry transition was weaker, i.e., not as jump-like as the collapse transition. These general features have recently been predicted by a mean-field type adsorption-attraction model, which can be a good starting point to qualitatively describe the cononsolvency transition of PNiPAAm brushes. Without a doubt, grafting density, molecular weight, and the molecular weight distribution had an important influence on the properties of polymer brushes. The theoretical model made clear predictions for the influence of the grafting density: Increasing the grafting density should have increased the deswelling ratio, but the grafting density only had a little effect on the solvent-composition location of the minimum of the brush height. In future, we will further study how these parameters affect the cononsolvency transition properties of polymer brushes. This will allow for a comparison of scaling predictions of the model, and for an analysis of the various molecular contributions to the cononsolvency effects. Furthermore, computer simulations can address more detailed properties, such as the density profile of monomers.

## Figures and Tables

**Table 1:** Dry layer parameters (number-average molecular weight *M_n_*, thickness *h*, grafting density *σ*, grafting distance *S*, and corresponding brush criteria) of polymer films, which were used in this study.

Polymer	*M_n_* (g/mol)	*h* (nm)	*σ* (chains/nm^2^)	σ** (chains/nm^2^)	*S* (nm)	*S*/(2*R_G_*)	*S*/(2*R_P_*)
PGMA	10–20,000	2.5	-	-	-	-	-
PNiPAAm	50,200	14.9	0.20	0.084	2.3	0.1	0.5

## Appendix A

### Appendix A.1. Raw Data of Swollen Thickness of PNiPAAm Brushes

**Table A1:** Raw data of swollen brush thickness of a PNiPAAm brush plotted in Figure 1d.

**Ethanol Concentration**	0 vol %	5 vol %	7 vol %	10 vol %	11 vol %	12 vol %	13 vol %
**Brush Thickness (nm)**	53.2	51.3	47.2	43.7	41.4	35.8	31.3

**Table A2:** Raw data of swollen brush thickness of a PNiPAAm brush plotted in Figure 1d.

**Ethanol Concentration**	14 vol %	15 vol %	16 vol %	17 vol %	18 vol %	19 vol %	20 vol %
**Brush Thickness (nm)**	27.1	26.8	26.5	26.6	27.4	26.3	26.5

**Table A3:** Raw data of swollen brush thickness of a PNiPAAm brush plotted in Figure 1d.

**Ethanol Concentration**	25 vol %	35 vol %	50 vol %	65 vol %	80 vol %	100 vol %
**Brush Thickness (nm)**	27.6	30.9	38.6	53.2	62.6	65.7

### Appendix A.2. The Role of Volume of Solvent Molecules in the Swelling of PNiPAAm Brushes

Based on scaling theory [6], the brush’s height, *H*, on a flat surface can be expressed as

(A1) $$H=kb^{\frac{1}{x}}S^{1-\frac{1}{x}}N,$$

where *b* is the actual monomer size, *S* is the average distance between two adjacent grafted polymer chains, *N* is the degree of polymerization, *k* is a numerical prefactor that depends on temperature and polymer’s chemical structure, and *x* is the Flory exponent.

The size of a water molecule is about 0.28 nm, the size of an ethanol molecule is about 0.44 nm, and the size of a PNiPAAm repeat unit is about 0.70 nm [42]. Here, we assume that in pure solvents, every repeat unit of PNiPAAm adsorbs a solvent molecule. Then, the actual monomer size of PNiPAAm in pure ethanol is about 1.14 nm (=0.70 nm + 0.44 nm), and the actual monomer size of PNiPAAm in pure water is about 0.98 nm (=0.70 nm + 0.28 nm). Then the ratio of brush height in pure ethanol *H_E_* to the brush height in pure water *H_W_*, is *H_E_/H_W_* = (1.14/0.98)^1/*x*^. If we choose *x* = 0.588 for good solvents, we get *H_E_/H_W_* = 1.30. Our experimental result of *H_E_/H_W_* was 1.24 (=65.7/53.2).

The difference between 1.30 and 1.24 is about 5%, therefore our theoretical calculation was consistent with our experimental results. Note that in our calculations, for pure solvents, we assume every repeat unit of PNiPAAm adsorbs a solvent molecule. This assumption implies that the brush thickness in pure ethanol (V_E_ = 1) is always thicker than in pure water (V_E_ = 0) due to the fact that molecular volume of ethanol is larger than water’s.

### Appendix A.3. Definition of Chemical Potential Change

Experimental chemical potential change used in this paper is defined as the same as in a standard Physical Chemistry book [43], namely:

(A2) $$\mu =\ln\left[\frac{\gamma_{E}x_{E}}{\gamma_{w}\left(1-x_{E}\right)}\right]$$

where *γ_E_* and *γ_w_* are activity coefficients for ethanol and water, respectively (they are functions of temperature and composition change), and *x_E_* is the molar fraction of ethanol in the ethanol-water mixture. The quantities *γ_E_* and *γ_w_* can be calculated by using the DDB Software Package [44].
